# Genomic changes and biochemical alterations of seed protein and oil content in a subset of fast neutron induced soybean mutants

**DOI:** 10.1186/s12870-019-1981-x

**Published:** 2019-10-12

**Authors:** Nazrul Islam, Robert M. Stupar, Song Qijian, Devanand L. Luthria, Wesley Garrett, Adrian O. Stec, Jeff Roessler, Savithiry S. Natarajan

**Affiliations:** 1 Soybean Genomics and Improvement Laboratory, USDA-ARS, NEA, 10300, Baltimore Avenue, Beltsville, MD USA; 2 Department of Agronomy and Plant Genetics, University of Minnesota, St. Paul, MN USA; 3 Food Composition and Methods Development Laboratory, USDA-ARS, NEA, Beltsville, MD USA; 4 Animal Biosciences & Biotechnology Laboratory, USDA-ARS, NEA, Beltsville, MD USA

**Keywords:** Soybeans, Proteins, Gene deletion, Fatty acids, Oil, Mutants, Comparative genomic hybridization, Protein

## Abstract

**Background:**

Soybean is subjected to genetic manipulation by breeding, mutation, and transgenic approaches to produce value-added quality traits. Among those genetic approaches, mutagenesis through fast neutrons radiation is intriguing because it yields a variety of mutations, including single/multiple gene deletions and/or duplications. Characterizing the seed composition of the fast neutron mutants and its relationship with gene mutation is useful towards understanding oil and protein traits in soybean.

**Results:**

From a large population of fast neutron mutagenized plants, we selected ten mutants based on a screening of total oil and protein content using near infra-red spectroscopy. These ten mutants were regrown, and the seeds were analyzed for oil by GC-MS, protein profiling by SDS-PAGE and gene mapping by comparative genomic hybridization. The mutant 2R29C14Cladecr233cMN15 (nicknamed in this study as L10) showed higher protein and lower oil content compared to the wild type, followed by three other lines (nicknamed in this study as L03, L05, and L06). We characterized the fatty acid methyl esters profile of the trans-esterified oil and found the presence of five major fatty acids (palmitic, stearic, oleic, linoleic, and linolenic acids) at varying proportions among the mutants. Protein profile using SDS-PAGE of the ten mutants did exhibit discernable variation between storage (glycinin and β-conglycinin) and anti-nutritional factor (trypsin inhibitor) proteins. In addition, we physically mapped the position of the gene deletions or duplications in each mutant using comparative genomic hybridization.

**Conclusion:**

Characterization of oil and protein profile in soybean fast neutron mutants will assist scientist and breeders to develop new value-added soybeans with improved protein and oil quality traits.

**Electronic supplementary material:**

The online version of this article (10.1186/s12870-019-1981-x) contains supplementary material, which is available to authorized users.

## Background

Genetic manipulation of soybean (*Glycine max [L.] Merr*) has been an area of renewed interest since the inception of the reference genome sequence in 2010 [1]. Because of the higher protein (~ 40%) and oil (~ 20%) content of soybean, it has been targeted by the food industry to produce a variety of nutritionally enhanced food products [2–4]. Several approaches have been utilized to manipulate the genome composition of soybean to produce desirable qualitative traits. This includes, but is not limited to, transposon tagging [5], chemical treatments [6], radiation mutagenesis [7], genetic transformation, and gene editing [4, 8]. Radiation mutagenesis, particularly fast neutron (FN) radiation, causes a wide range of variation through deletions, duplications, translocations, and inversions, which can induce strong mutant phenotypes [7, 9]. This area of interest has been further augmented with the increased capabilities of whole genome sequencing [7, 9]. Radiation mutagenesis deletes cluster of adjacent genes, including tandem repeats, and does not require the insertion of any foreign gene. Bolon et.al. [7] produced more than 27,000 unique soybean mutants using FN bombardment (https://www.soybase.org/mutants/about.php). Using array-comparative genomic hybridizations (CGHs) along with next-generation sequencing (NGS) technologies, the investigators unveiled the genome-wide structural variations in a subset of FN mutated soybeans [9]. Furthermore, distinct FN-induced sequence rearrangements at a NAP1 gene model associated with stunted trichrome development in soybean has also been reported [10]. Furthermore, an FN-induced reciprocal chromosomal translocation was found to underlie a mutant phenotype exhibiting high sucrose and low oil in seeds [11]. Stacey et al. [12] used FN mutagenesis to elucidate the functional network of a *GmHGO1* gene associated with homogentisate catabolism that lead to a brown seeded phenotype in soybean.

Currently, there are deletion/duplication profiles for several hundreds of FN mutant lines, which are a community resource for informative genome analyses [9]. However, to make the best use of the FN mutants, a comprehensive biochemical analysis of the mutants in relation to quality attributes is paramount. In this study, we screened a large population of FN mutants to identify a subset with large alterations in oil and protein profiling. A subset of ten mutants were subjected to a detailed seed composition analyses, including fatty acid methyl esters (FAMEs) composition of the transesterified oil and protein profile. In addition, we physically mapped the gene deletion/duplications caused by the mutagenesis. This information will aid breeders/biotechnologists to incorporate the desired high oil and protein traits and develop new value-added soybeans.

## Results

### Identification of mutant lines with altered seed composition traits

Mutants from the soybean FN population from genetic background M92–220 [11] were grown in the field (University of Minnesota, St. Paul, MN) for several years, allowing for successive rounds of self-pollination and stabilization of the phenotypes. A wide range of phenotypes was observed in the mutant population [7], and the surviving mutants produced healthy seeds for each successive generation. Field harvested seeds from several thousand FN lines in 2015 (University of Minnesota, St. Paul, MN) were initially screened by near-infrared spectroscopy (NIR) analysis to estimate the relative proportion of seed constituents (Table 1; Additional file 3: Figure S1). For this study, ten lines exhibiting outlier levels of seed protein and/or oil were selected for further analyses. The field-harvested seeds from the ten lines did not exhibit any obvious seed morphology phenotypes or differences in individual seed weight. The full names of these lines used in this study are shown in Table 1. However, for simplicity, the mutants have been nicknamed L01-L10 for the purposes of this manuscript.

**Table 1:** List of FN mutants and their NIR data from the 2015 field-harvested seeds

Mutants	Noted as	Protein (%)	Oil (%)	P + O
M92–220	Wild type	41.8	20.5	62.3
5R16C01Dacar261bMN15	L01	30.5	25.7	56.3
R29C32DSaeccdr240bMN15	L02	33.1	22.8	55.9
5R10C28Decfbar241aMN15	L03	55.2	13.4	68.6
P1016CaBr505dBMN15	L04	41.0	16.0	57.0
R60C28p35c02br177aMN15	L05	52.0	17.4	69.4
R32C17Dccbbcdar223bMN15	L06	51.3	14.6	65.9
2R29C14Ccar39aMN15	L07	40.7	21.7	62.4
2012CM7F040p05ar154bMN15	L08	47.8	15.0	62.8
R15C01P33a02ar168bMN15	L09	50.6	16.8	67.5
2R29C14Cladecr233cMN15	L10	58.0	10.9	69.0

For the ten selected mutant lines, the total seed protein content ranged between 30.5 to 58.0% and the total seed oil content varied between 10.9 to 25.7%. The total seed protein and oil (P + O) content in the ten mutant soybean seeds varied between 55.9 to 69.4%. The total protein, oil and P + O content in the wild type soybean seed was observed to be 41.8, 20.5, and 62.3% respectively. Six lines (L03, L05, L06, L08, L09, and L10) showed higher protein content when compared to the wild type (Table 1). However, four mutant lines L01 (30.5%), L02 (33.1%), L04 (41.0%) and L07 (40.7%) indicated lower protein content as compared to the wild type. Among the mutants, L10 exhibited the highest protein content (58.04%), L01 showed higher oil content (25.7%) and L05 showed highest P + O (69.4%) content.

### Fatty acid methyl Ester (FAMEs) analysis of transesterified oil extracted from FN mutants

**Fig. 1:**
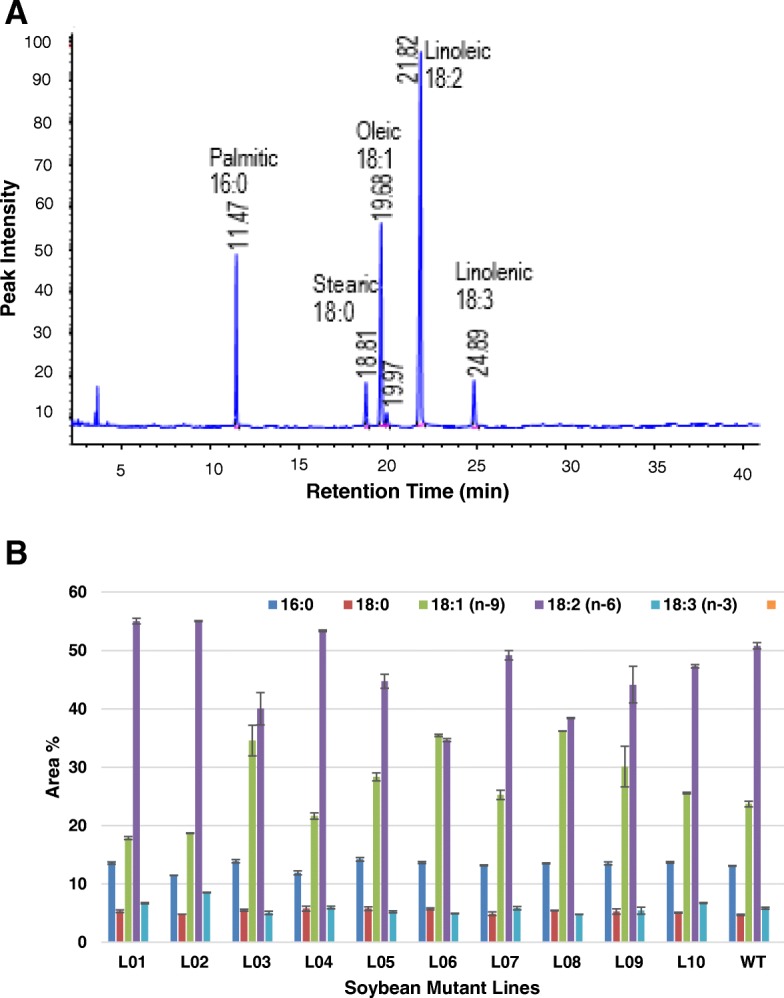
**a** A typical GC-FID profile of fatty acid methyl esters (FAMEs) of trans-esterified oil and **b** Fatty acids content of the FN mutants. The bar represents standard error

The ten parental mutants with varying protein and oil content were regrown in the field (University of Minnesota, St. Paul, MN) in 2016 to collect more seeds for analysis (Additional file 3: Figure S1). To estimate the fatty acid methyl ester profiling in the seeds, we employed a Gas Chromatography – Flame Ionization Detector (GC-FID). GC-FID analysis of the transesterified oil revealed the presence of five major fatty acid methyl esters (palmitic, stearic, oleic, linoleic, and linolenic acids). A typical GC-FID profile of the transesterified soybean oil sample is shown in Fig. 1a. As evident from the Fig. 1a, five major FAMEs were detected in the transesterified oil extracted from ten mutants, which accounted for over 95% of the total FAMEs. These were identified as methyl palmitate acid (16:0), methyl stearate acid (18:0), methyl oleate (18:1), methyl linoleate acid (18:2) and methyl linolenate acid (18:3). The average saturated fatty acid content (palmitic and stearic) in ten mutant samples varied between 16.3 to 20.0%, whereas the average unsaturated fatty acid content (oleic, linoleic and linolenic) in ten mutants varied between 75.0 to 82.3%. A similar saturated to unsaturated fatty acid (17.8 to 80.3%) ratio was observed in the wild type parent soybean M92–220 (Fig. 1b). Significant variations in individual FAMEs profiles were observed among the ten mutant lines. These variations were primarily in the oleic and linoleic acids content. The range for oleic acid varied between 17.9–36.2% whereas, the range for the linoleic acid varied between 34.6 and 55.0%. We observed a negative correlation between the oleic acid and linoleic acid content (R2 = − 0.96).

### Differentially expressed protein profile

**Fig. 2:**
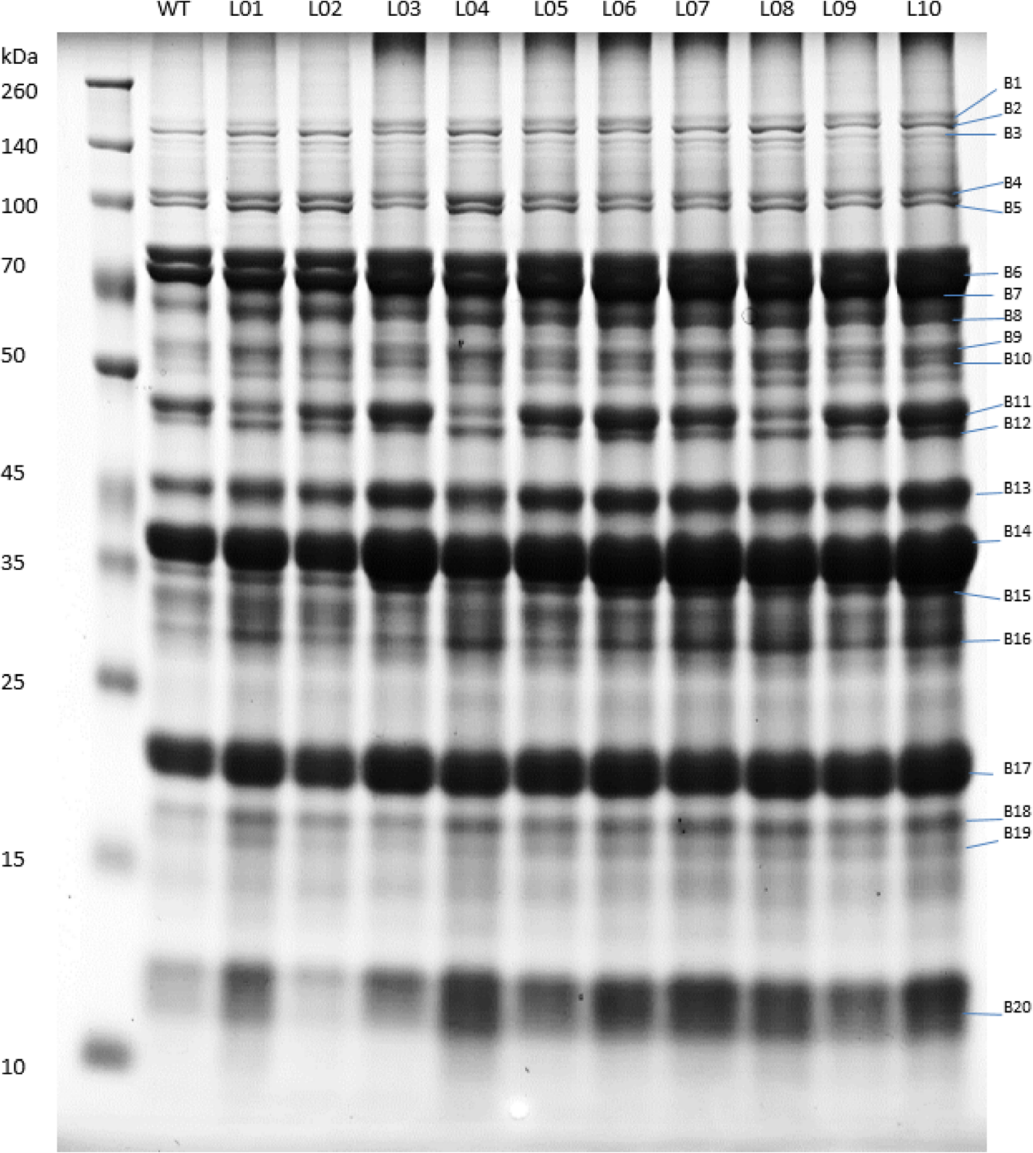
Protein profile of ten mutants as separated by SDS-PAGE. WT represents wild type; L01 to L10 represent mutants; B represents different protein bands; kDa represents the molecular weight of the protein band

The 2016 field-harvested seeds were also used for protein expression analysis (Additional file 3: Figure S1). The total seed protein profile of the ten mutants was analyzed by SDS polyacrylamide gel electrophoresis (Fig. 2). Variations in the protein profiles among the ten mutants were observed in storage and anti-nutritional proteins. Approximately 20 protein bands were conspicuously observed, some of which appeared to be differentially expressed based on relative abundance. The differentially expressed protein bands were manually excised and digested with trypsin. The resulting peptides were identified using MALDI/TOF/TOF-MS and the results are presented in Table 2 and Additional file 1: Table S1. The mutants L03, L06, and L10 showed higher abundance of several bands such as, B6 (β-conglycinin, alpha’ chain precursor), B13 (β-conglycinin, β chain), B14 (glycinin G2 precursor) and B17 (trypsin inhibitor subtype A precursor) as compared to the wild type soybean M92–220. Band B11, identified as β-conglycinin β chain precursor, exhibited higher abundances in mutants L03, L05, L06, L09 and L10. Similarly, B13, identified as β-conglycinin (β chain) showed higher abundances in all lines except L01, L02 and L04. The list of the peptides used to identify the proteins are shown in Additional file 1: Table S1 with protein accession number in addition to unique peptide spectral counts. Based on the molecular weight and banding patterns of the storage proteins (β-conglycinin and glycinin) and trypsin inhibitors, we subdivided them into different groups. The storage proteins in the ten mutants can be subdivided into three groups (group 1 – L01, L04, L08; group 2 – L02, L03, L05, L07, L09; and group 3 – L06 and L10). Glycinin A1b2B2–784 precursor (B20) was significantly lower in L02 than other mutant lines. Based on the trypsin inhibitors banding patterns (B16 and B17), the ten mutants subdivided into three groups, except L03 mutant (group 1- L07, L08, L10; group 2- L02, L05; group 3- L01, L04, L06, L09). Band-19 (trypsin inhibitor) showed low protein intensity and therefore could not be grouped.

**Table 2:** Identification of protein bands showed in the Fig. 2

Band	Accession	Protein name
B1	NP_001235827	glycinin G1 precursor
B2	XP_003556052	beta-conglycinin, beta chain
B3	NP_001235827	glycinin G1 precursor
B4	NP_001237316	beta-conglycinin, alpha’ chain precursor
B5	NP_001237316	beta-conglycinin, alpha’ chain precursor
B6	NP_001237316	beta-conglycinin, alpha’ chain precursor
B7	XP_014628505	beta-conglycinin, alpha chain-like
B8	NP_001235810	glycinin G2 precursor
B9	NP_001235827	glycinin G1 precursor
B10	NP_001235810	glycinin G2 precursor
B11	NP_001236872	beta-conglycinin, beta chain precursor
B12	NP_001235810	glycinin G2 precursor
B13	XP_003556052	beta-conglycinin, beta chain
B14/15	NP_001235810	glycinin G2 precursor
B16	NP_001235651	trypsin inhibitor subtype A precursor
B17	NP_001235651	trypsin inhibitor subtype A precursor
B18	NP_001236676	glycinin precursor
B19	NP_001235651	Trypsin inhibitors
B20	NP_001236840	glycinin A1bB2–784 precursor

### Gene deletion/duplication of the FN mutants

**Fig. 3:**
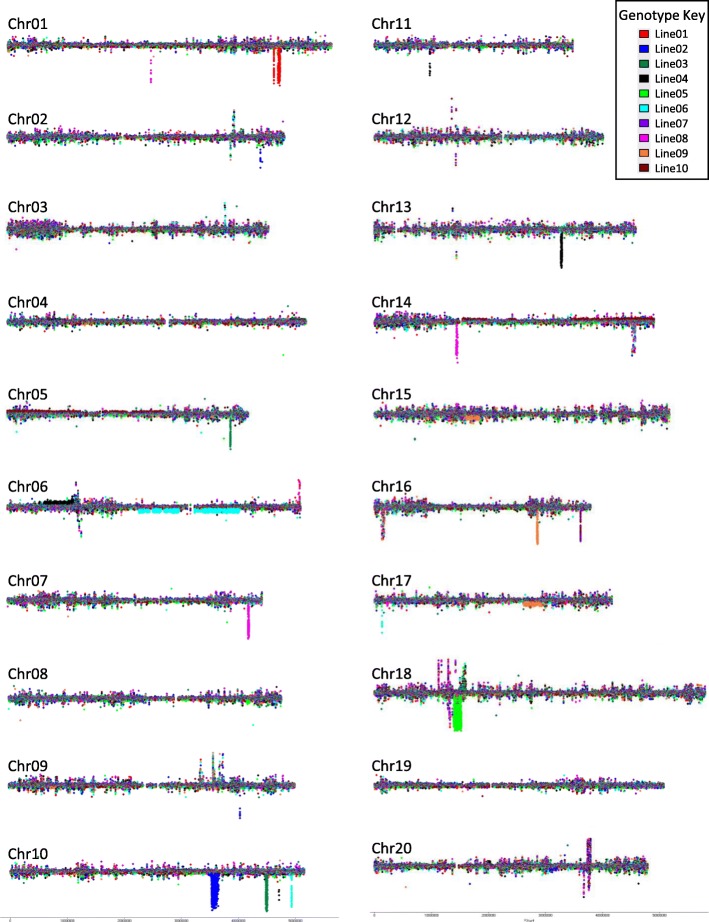
Overlapping CGH profiles of the 10 FN lines (colored by genotype) in this study across the 20 soybean chromosomes. The dominant horizontal line running through each chromosome represents a log_2_ ratio of zero (no difference) between the FN line and the control line (‘M92–220’). Peaks above the line represent likely duplications and peaks below the line represent likely deletions. Peaks in which multiple genotypes exhibit the change are oftentimes natural variants, while peaks exhibited by a single genotype are likely FN-induced

We utilized CGH analysis to identify and estimate the locations of gene deletions and duplications in the ten mutants compared to the wild type parental line M92–220. A single plant grown in a greenhouse in 2017 was used to represent each of the mutant lines (Additional file 3: Figure S1). The CGH profile of the ten mutants across the 20 chromosomes is presented in Fig. 3, and the raw data are available through the GEO accession number GSE118594. In these analyses, the horizontal line represents a log_2_ ratio of zero (no difference) for each chromosome between the FN line and the wild type line. Any signal in the vertical direction represents variation (likely duplications or deletions) between mutants. As evident from the Fig. 3, significant variations of gene duplication/deletion were observed across the ten mutant lines. The FN mutants along with the locations of deletions and duplications are presented in Table 3. The number of genes involved in the deletions and duplications are also shown in Table 3, and the gene model names are presented on Additional file 2: Table S2. In some lines, several hundred genes were deleted (homozygous or heterozygous)/duplicated due to the FN radiation. Some large heterozygous deletions were observed in some lines. For example, L06 showed the highest number of genes located within heterozygous deletions (246 genes) followed by L09 (103 genes). However, the amplitude of the CGH signals indicated that these were heterozygous deletions that would almost certainly render the organism unviable in homozygous deletion segregants. On the other hand, large duplications (encompassing a total of 1743 genes) were observed in L10. It is possible that large deletions occurring in regions with conserved intact paralogous genes elsewhere in the genome may have increased probabilities of survival in the homozygous deletion state. However, this circumstance would be expected to be rare in soybean, as the most recently duplication event occurred millions of years ago and most ancient homeologous regions are no longer highly conserved. On the other hand, large duplications may not have severe phenotypic consequences, and were in fact observed in some lines in this study. This was particularly true for L10, in which duplications encompassed a total of 1743 genes. The details of the genes that were deleted or duplicated due to the FN radiation are provided in the Additional file 2: Table S2.

**Table 3:** CGH analyses of Fast Neutron induced mutants

*Genotype*	*Chromosome*	*Start*	*End*	*Type of perturbation*	*Genes(no.)*
*L01*	Gm01	46707925	46750743	Homozygous Deletion	4
	Gm01	47452956	47801756	Homozygous Deletion	19
*L02*	Gm02	44345357	44350035	Homozygous Deletion	1
	Gm09	40575108	40579925	Homozygous Deletion	1
	Gm10	35254127	36582024	Homozygous Deletion	52
*L03*	Gm05	39061852	39117234	Homozygous Deletion	7
	Gm10	44814671	45068086	Homozygous Deletion	17
	Gm15	7051245	7051686	Homozygous Deletion	0
*L04*	Gm06	5606804	5656217	Duplication	6
	Gm06	6427918	11549153	Duplication	582
	Gm10	47113936	47115142	Homozygous Deletion	0
	Gm11	9716728	9722963	Homozygous Deletion	0
	Gm13	32702923	32899469	Homozygous Deletion	22
	Gm20	19893693	20291239	Duplication	6
*L05*	Gm13	13007316	13007806	Heterozygous Deletion	0
	Gm18	13845321	15237935	Homozygous Deletion	11
*L06*	Gm06	4238463	4238908	Heterozygous Deletion	0
	Gm06	23069004	30055434	Heterozygous Deletion	67
	Gm06	32783476	40641172	Heterozygous Deletion	179
	Gm10	49314172	49352798	Homozygous Deletion	4
	Gm17	1303183	1308540	Homozygous Deletion	0
*L07*	Gm16	36142905	36166915	Homozygous Deletion	2
*L08*	Gm01	25162702	25168882	Homozygous Deletion	0
	Gm07	42193407	42352938	Homozygous Deletion	18
	Gm14	14337661	14594509	Homozygous Deletion	3
*L09*	Gm15	16117931	18439687	Heterozygous Deletion	103
	Gm15	19631155	19681225	Heterozygous Deletion	0
	Gm16	28495594	28639238	Homozygous Deletion	5
	Gm17	26178653	29832257	Heterozygous Deletion	0
*L10*	Gm05	10338	12752724	Duplication	819
	Gm05	16673931	27307580	Duplication	157
	Gm14	15425024	19713778	Duplication	67
	Gm14	23375328	24823916	Duplication	34
	Gm14	34475571	49032182	Duplication	666
	Gm16	36142905	36166915	Homozygous Deletion	2

To confirm the gene deletions, we preformed PCR analysis. We selected at least one gene from each region of deletion and duplication for the homozygous plants. However, in some mutants such as L07, L08, and L09, the analyses couldn’t be performed because of the non-specificity of the primers or the poor/failure amplification. Based on the specificity, PCR analyses were performed for eight genes and the gel image is presented in the Additional file 4: Figure S2. As shown in the Figure, all the mutants did not exhibit the gene product or amplified except the L10 in which the selected gene was duplicated.

## Discussion

### Prospects for seed composition improvement through FN mutagenesis

We performed several analyses to characterize the soybean fast neutron mutants, including CGH to locate the gene deletion/duplication, NIR to estimate protein and oil levels, SDS PAGE for seed protein profiling, and GC-FID for FAMEs composition. The results presented in this manuscript demonstrate that a 5 to 15% (dry weight basis) increase in protein content in soybean seed is possible using fast neutrons radiation. Among the ten mutants tested (L01-L10), L10 showed a 58% total seed protein content when compared to the wild type parent M92–220 (41.8%). Soybean has the highest protein contents among other legumes, averaging approximately 40 to 42% (dry weight basis). However, an increase in protein content in the soybean seed is desirable, as the higher protein content increases the value of soybean. A study conducted by the center consulting group LLC concluded that, when yield and oil levels remain the same, a 1% increase in protein content increases a crop’s value per acre (https://www.agprofessional.com). As per the United Soybean Board (USB) (https://unitedsoybean.org), poultry and livestock farmers prefer soybean with high protein content.

Several approaches have been utilized to increase protein content in soybean seeds. In this endeavor, a genetic gains strategy through improved breeding practice is being adopted and a detailed molecular mapping of the genes associated with proteins has been documented [13]. As reported, chromosomes 20 (linkage group I (*LG*-*I*)), and 15 (*LG*-*E*) contain the major quantitative trait loci (QTL) for soybean protein variation [13]. However, a genetic gains strategy through improved breeding involves several challenges. For instance, the domesticated soybean is a paleopolyploid, and approximately 75% of the genes have multiple copies [1]. Therefore, deletion or addition of a targeted gene does not always provide the expected results. In addition, transgenic approaches have also been adopted to increase soybean protein content [14, 15]. In these investigations, a foreign gene was introduced to increase the level of protein in the soybean seed. However, this approach requires an expensive and prolonged regulatory approval to release the traits in the market place. FN based mutagenesis does not involve foreign gene introduction and thus does not require approval through the regulatory process.

While FN mutagenesis is clearly capable of creating large increases in total seed protein, such lines would need to be extensively evaluated for additional traits prior to commercialization. This would include agronomic traits, particularly yield. It would not be surprising to observe detrimental traits within a line that exhibits radically altered seed composition traits. Furthermore, it may be desirable to identify the specific deletions and/or duplications underlying the seed protein increase, as these could be backcrossed into elite varieties. If the FN seed composition locus also causes detrimental traits, it may not be useful for breeding per se. However, it may still be useful for identifying genes that control these traits, leading to targeted breeding strategies that utilize natural or other forms of induced variation for these genes.

### The relationship between seed protein, oil, and fatty acid compositions

We observed a negative correlation (r^2^ = − 0.8302) between the protein and oil composition of the mutants (Table 1). This result indicates that the FN-altered genomic changes that causes more protein content typically also results low total oil content, and vice versa. Results from other large scale investigations reported similar findings [16, 17], and it is generally accepted that soybean seed protein and oil are inversely correlated among natural variants, probably because of carbon distribution. Although the exact reason for such a negative correlation is not known, Wilson [18] suggested a model to overcome the barrier between protein and oil content by estimating constituent value. As suggested, based on average protein (42%) and oil (19%) content of soybean germplasm of the USDA collection, a pragmatic goal might be set for a variety with 44 to 45% protein and no less than 18% oil content. In our investigation, FN induction yielded a wide range of variation in protein (40 to 58%) and oil (10 to 25%). The present study showed that some FN lines have potential to improve the quality traits in soybeans as suggested by Wilson [18]. However, when considering these data, some additional factors must be accounted. For example, the mutants in this study were not tested for harvestable yield or other agronomic performance traits. As mentioned above, it is possible that the alterations to the seed composition traits, or other mutations in these plants, may influence other traits that are important to the growers.

Like the oil content, variations in fatty acid content of the trans-esterified oil extracted from the ten mutant soybeans were also investigated. Although L10 exhibited low total oil content, the fatty acid composition of all ten mutants and the wild type soybeans showed the presence of five prominent fatty acids, namely, palmitic, stearic, oleic, linoleic and linolenic acids. Similar fatty acid composition (13% of palmitic acid (16:0), 4% stearic acid (18:0), 20% oleic acid (18:1), 55% linoleic acid (18:2) and 8% linolenic acid (18:3)) has been previously reported in the literature [19, 20]. Among these, the palmitic and stearic acids are saturated fatty acids, and the remaining are unsaturated fatty acids.

The unsaturated fatty acid profiles showed significant variation among the ten mutant samples. The range for oleic acid varied between 17.9 to 36.2%, with L08 showing the highest quantity and L01 showing the lowest quantity. It has been reported in the literature by Bellaloui et al. [21] that the variation in oleic acids composition may be due to agricultural practices, including planting date, seeding rate and growing conditions. The authors suggested that temperature influenced the enzymes involved in biosynthesis of fatty acids during the seed fill stage. In the FN mutants, the range for linoleic acid varied between 34.6 to 55.0%, with L02 showing the highest quantity and L06 showing the lowest quantity. The mutant Line L06 also showed increased levels of oleic acid (34.6%) and comparatively lower levels of linolenic acid (4.9%). This mutant line could be a significant interest to breeders interested in developing new value-added soybean with significant improvement in oil profiles of nutritional significance. It is desirable to have a reduced concentration of polyunsaturated fatty acids (18:3) in the oil, as it reduces the shelf life due to oxidation which causes an unpleasant odor [22].

### Variations in protein and gene deletion/duplication profiles

Clear variations in the seed protein profiles of the ten FN mutants for abundant proteins were observed in this study, especially for storage proteins and trypsin inhibitors (Fig. 2). Interestingly, the higher abundance of bands such as B6 (β-conglycinin, α’ chain precursor), B11 (β-conglycinin, β chain precursor), B14 (glycinin G2 precursor) and B17 (trypsin inhibitor subtype A precursor) corresponds with the high protein FN mutants as discussed before. We therefore anticipate that the β-conglycinin may have contributed to the higher protein content in the seeds. Krishnan and Nelson (2011) investigated the protein content of nine soybean accessions and concluded that the total higher protein content was mostly contributed by globulin [23] which includes β-conglycinin and glycinin. However, it is not known in our study how the FN irradiation induced higher protein content in some of the soybean lines. We also checked the regions of deletion/duplication to assess the corresponding genes of the region. Several mutants did not show duplication of the storage protein genes. L10, however, exhibited duplication of genes encoding bifunctional inhibitor/lipid-transfer protein/seed storage 2S albumin superfamily protein. The 2S albumins, defined based on the sedimentation coefficient, are a group of storage proteins which contains sulphur containing amino acid [24, 25]. The 2S albumin also includes enzymatic proteins such as protease inhibitors which includes Bowman Birk and Kunitz trypsin inhibitors [26]. We, therefore, anticipate that deletion/duplication may have other pleotropic effects that contributed to the higher protein content in some of the FN mutants.

**Fig. 4:**
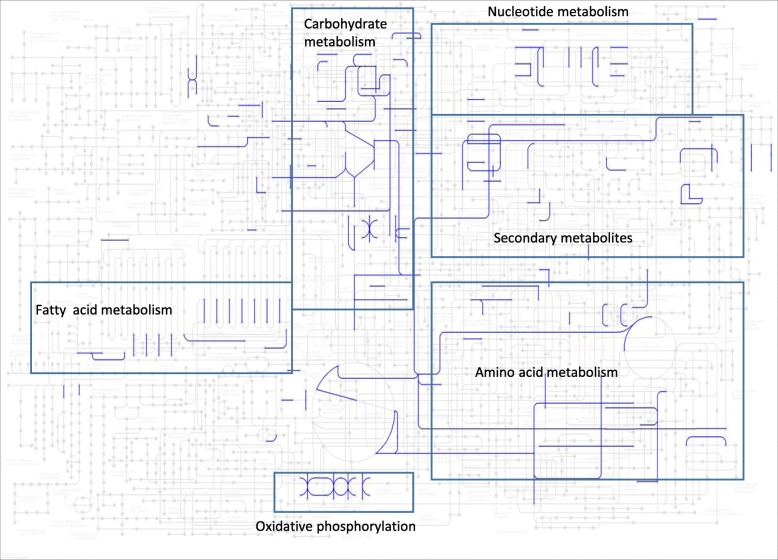
Mapping of duplicated genes in mutant L10 on the global metabolic pathways

To understand the potential mechanism causing the alterations of seed protein and oil, we mapped the duplicated genes for L10 on the global metabolic pathways. As evident from the Fig. 4, several pathways such as glycolysis / gluconeogenesis, fatty acid degradation, purine metabolism, biosynthesis of amino acids, ribosome, protein processing in endoplasmic reticulum, oxidative phosphorylation were enriched. The higher protein content of L10 is plausible from the enrichment of ribosome, protein processing in endoplasmic reticulum pathways. On the other hand, the lower oil content of the L10 could be related to the carbon distribution from fatty acid to protein synthetic processes. While global pathway analyses of the duplicated genes have provided changes in the pathways, it is not known whether the effect of protein increase or oil decrease act of one or a combination of several genes. To understand the gene effect, segregation analyses with targeted gene is underway.

In general, the CGH analysis revealed a wide range of deletions and duplications in the mutant lines. We confirmed the CGH results of selected genes which are deleted or duplicated using PCR analyses. The results are consistent with the CGH analyses (Additional file 4: Figure S2). Identifying the causative deletion/duplication underlying the seed composition changes would require further experiments, specifically co-segregation analysis in backcrossed or outcrossed populations [11]. Also, there are important limitations to using FN and CGH to identify causative alterations or genes underlying these traits. If the causative deletions/duplications are large, they may encompass numerous genes and make the identification of causative gene difficult. Also, CGH oftentimes does not resolve small deletions/duplications, nor does it detect inversions/translocations unless they alter DNA segment copy numbers. Furthermore, CGH does not perfectly resolve the exact breakpoints of large deletions/duplications. Therefore, the data provided in Table 3 and Additional file 2: Table S2 represent estimates of FN disruption to the genes/genome based primarily on large deletions and duplications. Lastly, given the later generation of these materials, it is presumed that most deletions and duplications are fixed as homozygous within the lineages. However, CGH shows that some deletions are heterozygous and therefore potentially still segregating within the population. However, CGH does not easily distinguish between homozygous from heterozygous duplications, therefore it is unclear which of these events may still be segregating within these populations.

## Conclusions

In this investigation, the genome composition of the M92–220 soybean genotype was manipulated by FN radiation mutagenesis. The mutants developed through this approach were found to be phenotypically stable at the M5-M8 generation. Ten FN mutants were selected for detailed analysis. A comprehensive analysis of seed composition attributes, such as oil and protein content, were performed. A wide range of variation for protein and oil content was observed among the mutants. In addition, the locations and number of genes deleted/duplicated by the FN mutagenesis were also estimated from whole genome CGH analysis. This information and the mutants are useful for scientist and breeders to alter seed composition traits to produce value-added soybeans.

## Methods

### Mutant materials and initial seed composition screening

Development of the FN mutant population and FN radiation doses were done at McClellan Nuclear Radiation Center at the University of California-Davis and described in previous publications [7, 9]. All of the mutants were developed in the soybean line ‘M92–220,’ which was derived from the seed stock of cultivar ‘MN1302’ [27]. A large screening of field-harvested seeds from 2015 was subjected to seed composition profiling with NIR spectroscopy [7]. A subset of mutants with large changes in protein and oil levels were identified. These ten mutants were grown in the field conditions (St. Paul, MN) in 2016 and harvested seeds were subjected to composition analysis (see methods below). A list of the FN mutants used in this investigation is listed in the Table 1. The generation of the FN mutants ranged from M5 to M9.

### Fatty acid methyl ester analysis

Oil extraction from the ground soybean seed powder (100 mg) was extracted twice with hexane (5 mL) in an ultrasonic bath (power 600 watts) for a period of 15 min. The extracts were centrifuged at 5000 rpm for 10 min and the supernatant was collected in a separate vial. The residue was re-extracted with 5 mL fresh hexane. The pool supernatant was evaporated to dryness under slow stream of nitrogen gas. The concentrated soybean oil was re-suspended with 2 mL hexane. A partial aliquot of one mL was evaporated to dryness and transesterified to FAMEs using 5 mL of acidified methanol (10 mL of acetyl chloride to 90 mL of cold methanol). The mixture was stirred at ambient temperature overnight under inert nitrogen atmosphere. To the above mixture, 3 mL of water was added and the fatty acid methyl esters were extracted with 2 mL of hexane. The hexane layer was separated and analyzed with GC [28]. Fatty Acid Methyl Ester were characterized by comparison of detention time with an authentic FAMEs standard. All analyses are conducted in triplicates and standard errors are calculated.

### Protein extraction

Proteins were extracted using a phenol extraction protocol [29]. Briefly, 200 mg of the ground seed from each mutant line were initially defatted using hexane [30]. Approximately 1 mL of the extraction buffer containing sucrose (0.7 M), tris(0.5 M), EDTA (50 mM), KC l(0.1 M), DTT (25 mM) and PMSF (2 mM) was added and the mixture was incubated for 30 min at ambient room temperature with shaking. The supernatant was collected after centrifugation (8000 g) for 30 min. An equal amount of water saturated phenol was added to the supernatant and the sample was mixed well for 10 min and centrifuged (30 min at 4 °C). The proteins from the phenol phase was precipitated by 0.1 M ammonium acetate in methanol and incubated at -20 °C overnight. Protein pellets were collected after centrifugation at 15000 g for 30 min followed by washing with cold acetone for three times. The protein pellets were re-suspended in 6 M urea, 100 mM Tris-HCl and the concentration was estimated by bicinchoninic acid assay (Pierce, Rockford, IL). All analyses and extractions were performed in three replicates.

### Protein separation by SDS PAGE

Polyacrylamide gel electrophoresis was performed to separate proteins. Briefly, 10 μg protein per well was loaded in 15% (w/v) polyacrylamide-gels and separation was achieved at 100 V for 45 min using a Tris/glycine/sodium dodecyl sulfate buffer (Mini-Protean Tetra system, Bio-Rad, Hercules, CA). The molecular weights of proteins were estimated using the Fermentas Spectra Multicolor Low Range Protein Ladder (Thermo Fisher Scientific, Waltham, MA). The gel was stained using Bio-Safe Coomassie G-250 (Bio-Rad).

### Protein digestion and identification

Protein bands were excised from the Commassie stained gel and digested with porcine trypsin (Promega). The resulting peptides were analyzed with a AB SCIEX TOF/TOF 5800 MALDI-MS system using a Mascot Distiller ver. 2.3.0.0 (www.matrixscience.com). Protein identification was performed using the Mascot search engine (http://www.matrixscience.com), which uses a probability based scoring system. The NCBInr database was used for the peptide interrogation. The parameters for database searches with MS/MS spectra were as follows: Fragment Tolerance: 0.60 Da (Monoisotopic), Parent Tolerance: 50 PPM (Monoisotopic), Fixed Modifications: + 57 on C (Carbamidomethyl), Variable Modifications: − 18 on n (Glu- > pyro-Glu), − 17 on n (Gln- > pyro-Glu), + 16 on M (Oxidation), Max Missed Cleavages: 1.

### CGH analysis

Soybean plants were grown in the greenhouse in 2017 (Additional file 3: Figure S1). Young leaf tissues were harvested and DNA extracted using the Qiagen DNeasy method for downstream CGH analysis. CGH was performed as described previously [11]. The CGH microarray (Agilent Technologies, Santa Clara, CA, USA) includes over 940,000 probes, which can be accessed in accession number GPL22907 in the National Center for Biotechnology Information Gene Expression Omnibus (GEO) (http://www.ncbi.nlm.nih.gov/geo). This microarray platform essentially tiles the soybean genome, with greater probe densities within genic regions. CGH hybridization conditions, scanning and data analysis all followed the previously described methods [11]. These segments of deletion or duplication were analyzed by jbrowse (https://phytozome.jgi.doe.gov/jbrowse/index.html) of phytozome 12 using *Glycine max* Wm82 a2v1 to retrieve the corresponding genes.

### PCR analysis

Genes were selected from the homozygous deletion regions except L10 where a gene was selected from the duplicated region to use as a control. The primers were designed using NCBI primer-blast https://www.ncbi.nlm.nih.gov/tools/primer-blast/ against the *Glycine max* whole genome sequence. The primer sequences were then examined for specificity in the soybean genome using e-PCR program, only the primer sets with sequence specific in the deletion regions were used for deletion verification. The primer sequences were synthesized by IDT (https://www.idt.com/). The following primers were used for PCR analysis. L01,5′-GCATATGCTGATTGGTGGCAA-3′ (forward) and 5′-TTCCATGAGAAAGGGGTGCC-3′ (reverse) (Glyma.01G139500); L02, 5′- ACCAATGCTCCTCCGCATTT-3′ (forward) and 5′-TCTGTGGCAGTCAACAGAGT-3′ (reverse) (Glyma.09G180800); L03, 5′-ACCAATACTGACTTTTGATTCCCT-3′ (forward) and 5′-TGAAAGGGATGGCTCGGATG-3′ (reverse)(Glyma.05G208700); L04, 5′- TGTCCACTGTCCAGTTGTGAT-3′ (forward), and 5′-CCTTGGGCTTGCCTGAAGTT-3′ (reverse) (Glyma.13G213700); L04, 5′-TGCATTGCACTGTCATTACCC-3′ (forward) and 5 ‘-GCATGGCAAGCCGAAACTTA-3’ (reverse) (Glyma.13G215400); L05, 5′- GTGGCAACAGTGTGCTTAGG-3′ (forward) and 5′-AATCCAGTCTGCCCCTCTCT-3′ (reverse) (Glyma.18G117500); L06, 5′-TATGGTACCTCAGGCGGACA-3′ (forward) and 5′-TGTTGTGTGTCAAGTAGGGTT-3′ (reverse) (Glyma.10G271200); L10, 5′-GGTGGCAGCTATACAGCACT-3′ (forward) and 5 ‘-ACCTTAATTCAGACACTCTCAAGGA-3’ (reverse) (Glyma.05G002600).

PCR mixture containing 150 ng of DNA, 4 μM of forward and reverse primers, 250 μM of each nucleotide, 1X PCR Buffer, and *Taq* DNA polymerase in a total volume of 15 μL were heated for 3 min at 94 °C before PCR cycling. The PCR cycles consist of 40 s denaturation at 94 °C, 30 s annealing at 52 °C, and 40 s extension at 72 °C for 35 cycles, followed by a 10-min extension at 72 °C. PCR products were analyzed on a 2.0% agarose gel and was stained by ethidium bromide (0.5 μg/ml) and the gel image was captured.

## Additional files

Additional file 1: **Table S1**. Peptide spectral used to identify the proteins. (RTF 401 kb)

Additional file 2: **Table S2**. A list of genes deletion and or duplication in the ten mutants. (XLSX 173 kb)

Additional file 3: **Figure S1**. Years and environments for tissues harvested for each experiment in this study. (PPTX 38 kb)

Additional file 4: **Figure S2**. Agarose gel electrophoresis of the PCR product of genes deleted or duplicated between wild type and the mutants. WT, wild; L01-L10, mutant lines; M, 100-base pairs (bp) marker. (JPG 91 kb)

## Data Availability

All data generated or analyzed during this study are included in this published article and its Additional files.

## Abbreviations

CGHs: Comparative genomic hybridizations
FAME: Fatty acid methyl ester
FN: Fast neutron
GC-FID: Gas chromatography – flame ionization detector
MALDI: Matrix-assisted laser desorption/ionization
MS: Mass spectrometry
NGS: Next-generation sequencing
PCR: Polymerase chain reactions
QTL: Quantitative trait loci
SDS-PAGE: Sodium dodecyl sulfate polyacrylamide gel electrophoresis
TOF: Time-of-flight mass spectrometry
