# Supplementary material for: Genomic changes and biochemical alterations of seed protein and oil content in a subset of fast neutron induced soybean mutants
Source: BMC Plant Biol. 2019 Oct 12;19:420. doi: 10.1186/s12870-019-1981-x (PMC6790046; doi:10.1186/s12870-019-1981-x)
Supplement: Supplementary file 1 — Table S1. Peptide spectral used to identify the proteins. (RTF 401 kb) [file 12870_2019_1981_MOESM1_ESM.rtf]

Table S1:  Unique peptide spectral counts used to identify the proteins
Band No	Protein accession numbers	Peptide detected	Exclusive unique spectrum count	Protein name	
B1	NP_001235827	VFDGELQEGR	2	glycinin G1 precursor	
 	XP_014628505	EQQQEQQQEEQPLEVR	8	beta-conglycinin, alpha chain	
 	 	VPSGTTYYVVNPDNNENLR	8	 	
B2	XP_003556052	QVQELAFPGSAQDVER	1	beta-conglycinin, beta chain	
 	NP_001236840	NNNPFSFLVPPKESQR	1	glycinin A1bB2-784 precursor	
 	NP_001236872	QVQELAFPGSAQDVER	1	beta-conglycinin, beta chain precursor	
 	NP_001237316	VPAGTTYYVVNPDNDENLR	1	beta-conglycinin, beta chain precursor	
 	NP_001235827	VFDGELQEGR	2	glycinin G1 precursor	
 	 	VLIVPQNFVVAAR	2	 	
 	NP_001235795	AIPSEVLAHSYNLR	1	glycinin G4 precursor	
 	XP_014628505	EQQQEQQQEEQPLEVR	8	beta-conglycinin, alpha chain	
 	 	NPFLFGSNR	8	 	
 	 	VPSGTTYYVVNPDNNENLR	8	 	
B3	NP_001235827	VLIVPQNFVVAAR	2	glycinin G1 precursor	
 	XP_014628505	EQQQEQQQEEQPLEVR	8	beta-conglycinin, alpha chain	
B4	NP_001237316	VPAGTTYYVVNPDNDENLR	1	beta-conglycinin, alpha' chain precursor	
 	NP_001235827	VFDGELQEGR	2	glycinin G1 precursor	
 	 	VLIVPQNFVVAAR	2	 	
 	NP_001236153	QGEVFYVPR	1	seed linoleate 13S-lipoxygenase-1	
 	XP_014628505	EQQQEQQQEEQPLEVR	8	 beta-conglycinin, alpha chain	
 	NP_001235383	FQTLIDLSVIEILSR	1	seed linoleate 9S-lipoxygenase-3 	
B5	NP_001237316	VPAGTTYYVVNPDNDENLR	1	beta-conglycinin, alpha' chain precursor	
 	NP_001235827	VFDGELQEGR	2	glycinin G1 precursor	
 	 	VLIVPQNFVVAAR	2	 	
B6	NP_001237316	AIVVLVINEGEANIELVGIK	3	beta-conglycinin, alpha' chain precursor 	
 	 	FESFFLSSTQAQQSYLQGFSK	3	 	
 	 	VPAGTTYYVVNPDNDENLR	1	 	
 	 	TISSEDKPFNLR	1	 	
 	 	VPAGTTYYVVNPDNDENLR	1	 	
 B7	NP_001236856	FESFFLSSTEAQQSYLQGFSR	1	beta-conglycinin alpha-subunit precursor 	
 	 	FFEITPEKNPQLR	1	 	
 	 	QFPFPRPPHQK	1	beta-conglycinin alpha-subunit precursor 	
 	 	TISSEDKPFNLR	1	 beta-conglycinin, alpha chain-like	
 	 	VPSGTTYYVVNPDNNENLR	8		
 	XP_014628505	EQQQEQQQEEQPLEVR	8	 beta-conglycinin, alpha chain-like	
 	 	FESFFLSSTEAQQSYLQGFSR	1		
 	 	FFEITPEKNPQLR	1		
 	 	NKNPFLFGSNR	 	 	
 	 	NPFLFGSNR	8		
 	 	QFPFPRPPHQK	1	 	
 	 	TISSEDKPFNLR	1	 	
 	 	VPSGTTYYVVNPDNNENLR	8	 	
 	NP_001235810	NNNPFSFLVPPQESQR	1	 	
B8	NP_001237244	FEEFFGPGGR	2	sucrose-binding protein 2 precursor	
 	 	LLQGIENFR	2	sucrose-binding protein 2 precursor	
 	NP_001237316	TISSEDKPFNLR	1	beta-conglycinin, alpha' chain precursor	
 	 	VPAGTTYYVVNPDNDENLR	1	beta-conglycinin, alpha' chain precursor	
 	NP_001235827	VFDGELQEGR	2	glycinin G1 precursor	
 	XP_014628505	EEGQQQGEQR	8	beta-conglycinin, alpha chain	
 	 	EQQQEQQQEEQPLEVR	8		
 	 	GSEEEDEDEDEEQDER	8		
 	 	KQEEDEDEEQQR	8	 	
 	 	NPFLFGSNR	8		
 	 	SPQLQNLR	8	 	
 	 	TISSEDKPFNLR	1	 	
 	 	VPSGTTYYVVNPDNNENLR	8	 	
 B9	NP_001235827	VFDGELQEGR	2	 	
 	 	VLIVPQNFVVAAR	2	glycinin G1 precursor	
 	NP_001236872	AIVILVINEGDANIELVGIK	4	beta-conglycinin, beta chain precursor 	
 	 	EDENNPFYFR	3	 	
 	 	QVQELAFPGSAQDVER	1		
 	 	VREDENNPFYFR	4	 	
 	NP_001236856	AIVILVINEGDANIELVGLK	1	 beta-conglycinin alpha-subunit precursor	
 	 	FESFFLSSTEAQQSYLQGFSR	1		
 	 	FFEITPEKNPQLR	1		
 	 	VPSGTTYYVVNPDNNENLR	8	 	
 	XP_014628505	EQQQEQQQEEQPLEVR	8	beta-conglycinin, alpha chain-like	
 	 	FESFFLSSTEAQQSYLQGFSR	1		
 	 	FFEITPEKNPQLR	1		
 	 	VPSGTTYYVVNPDNNENLR	8	 	
 	NP_001235810	NNNPFSFLVPPQESQR	1	 	
B10	NP_001235827	VFDGELQEGR	2	glycinin G2 precursor	
 	XP_014628505	EQQQEQQQEEQPLEVR	8	beta-conglycinin, alpha chain	
 	NP_001236872	QVQELAFPGSAQDVER	1	beta-conglycinin, beta chain precursor 	
B11	NP_001236856	FFEITPEKNPQLR	1	beta-conglycinin alpha-subunit precursor 	
 	XP_014628505	FFEITPEKNPQLR	1	beta-conglycinin, alpha chain-like 	
 	NP_001235810	NNNPFSFLVPPQESQR	1	glycinin G2 precursor	
B12	NP_001237244	FEEFFGPGGR	2	sucrose-binding protein 2 precursor	
 	NP_001235827	VFDGELQEGR	2	sucrose-binding protein 2 precursor	
 	XP_014628505	EQQQEQQQEEQPLEVR	8	 beta-conglycinin, alpha chain	
 	 	NPFLFGSNR	8	 	
 B13	XP_003556052	EDENNPFYLR	1	beta-conglycinin, beta chain	
 	 	QVQELAFPGSAQDVER	1	 	
 	NP_001236872	EDENNPFYFR	3	beta-conglycinin, beta chain precursor	
 	 	QVQELAFPGSAQDVER	1	 	
 	 	VLFGEEEEQR	3	 	
 	 	VFDGELQEGR	2		
 	 	VLIVPQNFVVAAR	2		
 	XP_014628505	EEGQQQGEQR	8	 beta-conglycinin, alpha chain	
 	 	EQQQEQQQEEQPLEVR	8		
 	 	NPFLFGSNR	8		
 	 	VPSGTTYYVVNPDNNENLR	8		
 B14	NP_001235810	EAFGVNMQIVR	3	 glycinin G2 precursor	
 	 	FYLAGNQEQEFLK	3		
 	 	RFYLAGNQEQEFLK	3		
 	NP_001235810	NNNPFSFLVPPQESQR	1	 	
B15	NP_001236676	FNECQLNNLNALEPDHR	3	glycinin precursor	
 	 	HFLAQSFNTNEDTAEK	3	glycinin precursor	
 	NP_001236129	VPTVDVSVVDLTVR	1	glyceraldehyde-3-phosphate dehydrogenase	
 	NP_001237316	VPAGTTYYVVNPDNDENLR	1	beta-conglycinin, alpha' chain precursor	
 	NP_001235827	VFDGELQEGR	2	glycinin G1 precursor	
 	 	VLIVPQNFVVAAR	2	glycinin G1 precursor	
 	NP_001235795	AIPSEVLAHSYNLR	1	glycinin G1 precursor	
 	XP_014628505	EQQQEQQQEEQPLEVR	8	 beta-conglycinin, alpha chain	
 	 	NPFLFGSNR	8	 	
 	NP_001235651	GIGTIISSPYR	2	trypsin inhibitor subtype A precursor 	
B16	NP_001235810	NNNPFSFLVPPQESQR	1	glycinin G2 precursor	
 	NP_001236676	AIPSEVLSNSYNLGQSQVR	3	glycinin precursor	
 	NP_001237316	TISSEDKPFNLR	1	beta-conglycinin, alpha' chain precursor 	
 	NP_001235827	VFDGELQEGR	2	glycinin G1 precursor	
 	 	VLIVPQNFVVAAR	2	glycinin G1 precursor	
 	NP_001235795	AIPSEVLAHSYNLR	1	glycinin G4 precursor	
 	XP_014628505	TISSEDKPFNLR	1	beta-conglycinin, alpha chain	
 	 	VPSGTTYYVVNPDNNENLR	8		
 	NP_001235651	IGENKDAMDGWFR	2	trypsin inhibitor subtype A precursor 	
B17	NP_001236676	AIPSEVLSNSYNLGQSQVR	3	glycinin precursor	
B18	NP_001236840	NNNPFSFLVPPKESQR	1	glycinin A1bB2-784 precursor	
 	NP_001234972	HHGTTGVYGIDTDR	1	dehydrin-like protein [Glycine max]	
 	NP_001235827	VFDGELQEGR	2	glycinin G1 precursor	
 	 	VLIVPQNFVVAAR	2		
 	XP_014628505	EQQQEQQQEEQPLEVR	8	 beta-conglycinin, alpha chain	
 B19	NP_001235651	DAMDGWFR	2	uncharacterized protein	
 	 	GIGTIISSPYR	2		
 	NP_001236840	NNNPFSFLVPPKESQR	1	 glycinin A1bB2-784 precursor	
B20	NP_001235827	VFDGELQEGR	2	glycinin G1 precursor	
 	 	VLIVPQNFVVAAR	2	glycinin G1 precursor	
 	XP_014628505	EQQQEQQQEEQPLEVR	8	beta-conglycinin, alpha chain	
