# Supplementary material for: Genomic changes and biochemical alterations of seed protein and oil content in a subset of fast neutron induced soybean mutants
Source: BMC Plant Biol. 2019 Oct 12;19:420. doi: 10.1186/s12870-019-1981-x (PMC6790046; doi:10.1186/s12870-019-1981-x)
Supplement: Supplementary file 3 — Figure S1. Years and environments for tissues harvested for each experiment in this study. (PPTX 38 kb) [file 12870_2019_1981_MOESM3_ESM.pptx]

## Slide 1
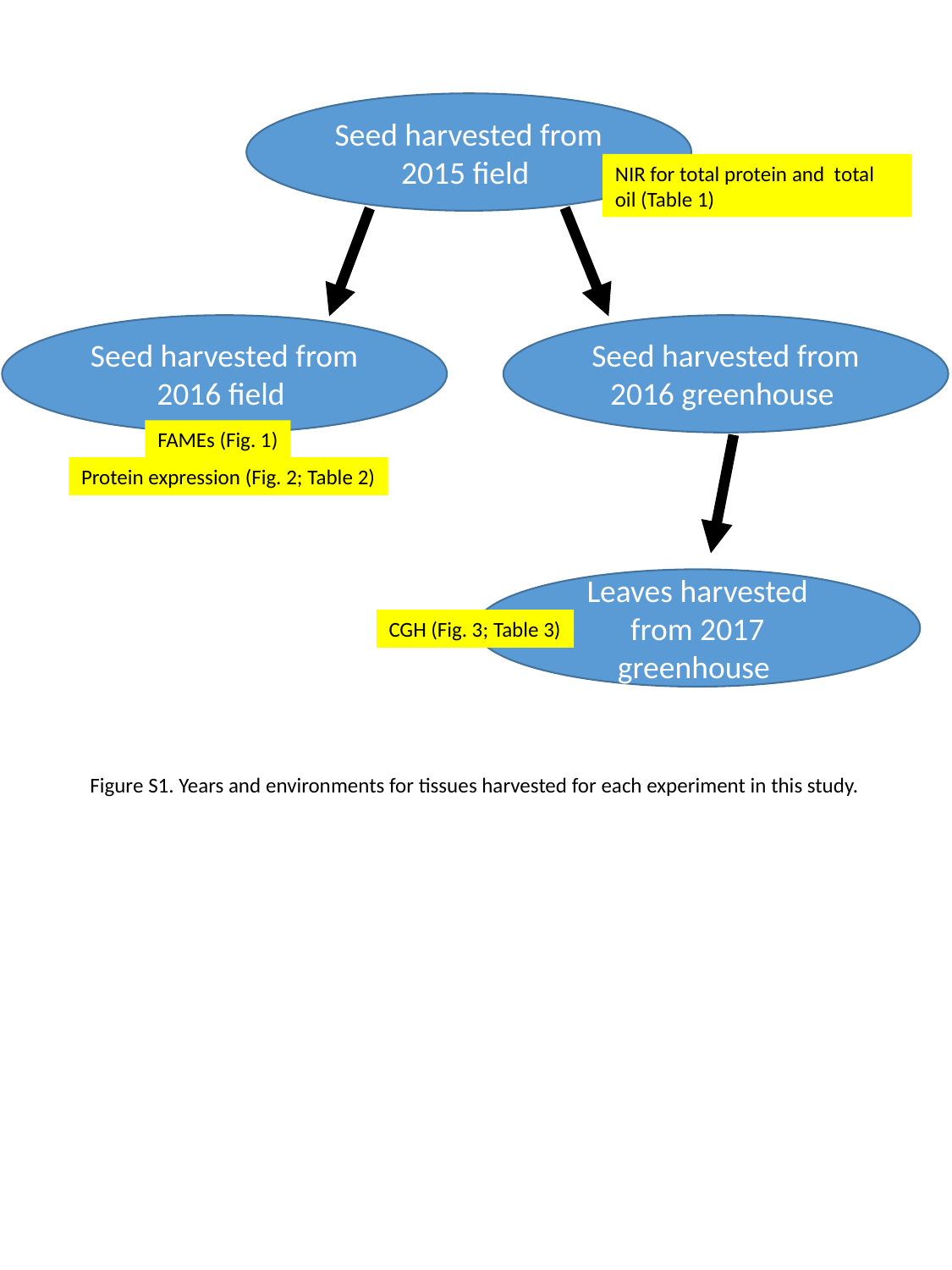

Seed harvested from 2015 field
NIR for total protein and total oil (Table 1)
Seed harvested from 2016 field
Seed harvested from 2016 greenhouse
FAMEs (Fig. 1)
Protein expression (Fig. 2; Table 2)
Leaves harvested from 2017 greenhouse
CGH (Fig. 3; Table 3)
Figure S1. Years and environments for tissues harvested for each experiment in this study.
