# Supplementary figures and images for: Genomic changes and biochemical alterations of seed protein and oil content in a subset of fast neutron induced soybean mutants
Source: BMC Plant Biol. 2019 Oct 12;19:420. doi: 10.1186/s12870-019-1981-x (PMC6790046; doi:10.1186/s12870-019-1981-x)

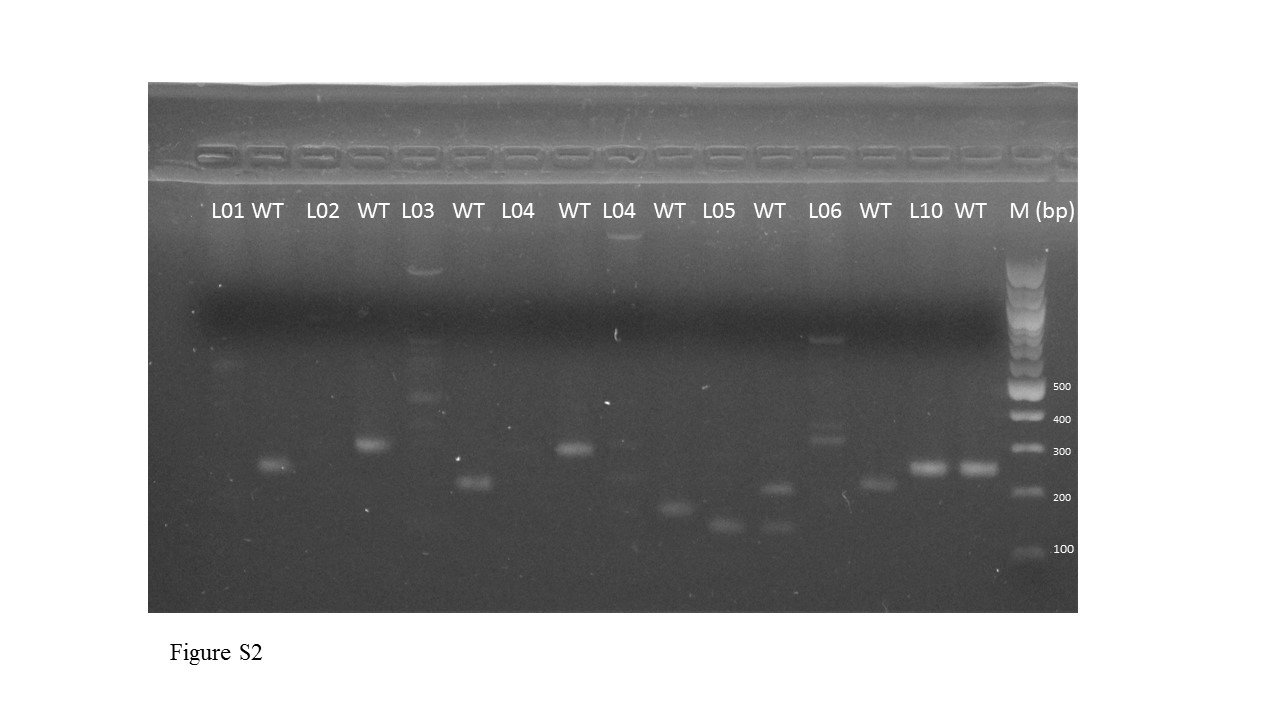

Supplement: Supplementary file 4 — Figure S2. Agarose gel electrophoresis of the PCR product of genes deleted or duplicated between wild type and the mutants. WT, wild; L01-L10, mutant lines; M, 100-base pairs (bp) marker. (JPG 91 kb) [file 12870_2019_1981_MOESM4_ESM.jpg]
